# Emerging superconductivity hidden beneath charge-transfer insulators

**DOI:** 10.1038/srep02235

**Published:** 2013-07-26

**Authors:** Yoshiharu Krockenberger, Hiroshi Irie, Osamu Matsumoto, Keitaro Yamagami, Masaya Mitsuhashi, Akio Tsukada, Michio Naito, Hideki Yamamoto

**Affiliations:** 1 NTT Basic Research Laboratories, NTT Corporation, 3-1 Morinosato-Wakamiya, Atsugi, Kanagawa 243-0198, Japan; 2 Department of Applied Physics, Tokyo University of Agriculture and Technology, 2-24-16 Naka-cho, Koganei, Tokyo 184-8588, Japan; 3 On leave from Nagaoka University of Technology.; 4 Current address: Department of Applied Physics, Tokyo University of Science, 1–3 Kagurazaka, Shinjuku, Tokyo 162-8601, Japan.

## Abstract

In many of today's most interesting materials, strong interactions prevail upon the magnetic moments, the electrons, and the crystal lattice, forming strong links between these different aspects of the system. Particularly, in two-dimensional cuprates, where copper is either five- or six-fold coordinated, superconductivity is commonly induced by chemical doping which is deemed to be mandatory by destruction of long-range antiferromagnetic order of 3*d*^9^ Cu^2+^ moments. Here we show that superconductivity can be induced in Pr_2_CuO_4_, where copper is four-fold coordinated. We induced this novel quantum state of Pr_2_CuO_4_ by realizing pristine square-planar coordinated copper in the copper-oxygen planes, thus, resulting in critical superconducting temperatures even higher than by chemical doping. Our results demonstrate new degrees of freedom, i.e., coordination of copper, for the manipulation of magnetic and superconducting order parameters in quantum materials.

While the issue of the mechanism of high temperature superconductivity continues to be controversial, one can clearly state that there have been many experimental results demonstrating that the lattices make a strong impact on the behavior of electrons^1^. First principle methods predict that square planar coordinated cuprates, e.g., Pr_2_CuO_4_ are expected to be metals^2^,^3^,^4^, unlike octahedral-coordinated cuprates. Square planar coordinated cuprates are those which, upon electron doping, become eventually high temperature superconductors subject to an annealing treatment^5^. The purpose of the annealing process is not related to an improved crystal quality but an adjusted oxygen stoichiometry irrespective of the Ce concentration *x*. A rich interplay of magnetic and electronic phases are reported^6^,^7^,^8^,^9^ for electron doped cuprates in relation to the doping concentration *x*. In particular, the vicinity of the insulating antiferromagnetic ground state to the superconducting ground state has been investigated^8^,^10^,^11^,^12^ and it was found that both phases, superconducting and insulating, are in competition with each other. Moreover, the boundary between the superconducting and antiferromagnetic-insulating phases in the electronic phase diagram of electron doped cuprate superconductors is not associated to a definite value *x*, but rather varies. Li *et al*.^13^ and Charpentier *et al.*^14^ reported a critical doping concentration *x*_c_ = 0.12, Krockenberger *et al.*^15^ reported *x*_c_ = 0.10 and Brinkmann *et al*. reported a *x*_c_ = 0.04 16 for superconducting Pr_2-x_Ce_x_CuO_4_. Furthermore, Matsumoto *et al*.^17^ reported superconductivity even for *x_c_* = 0.00 in Nd_2-x_Ce_x_CuO_4_. Although each group applies its unique annealing recipe, it is common to all five reports that the annealing conditions themselves have been kept almost constant over the entire doping range. The wide range of *x*_c_ between 0.00 and 0.12 suggests that the annealing recipe affects the competition between the antiferromagnetic insulating and superconducting states, severely. Additionally, the wide range of *x*_c_ may reflect that the appropriate annealing conditions for the induction of superconductivity themselves are doping dependent.

Electron-doped cuprate superconductors adopt the T′-structure (Nd_2_CuO_4_ structure) where two primary sites are occupied by oxygen: O(1) in the CuO_2_ planes and O(2) in the rare-earth (RE) oxide layers. Apical oxygen should not exist in the ideal T′ structure though they are clearly observed by Raman and far-infrared crystal-field spectroscopy^18^,^19^, Mössbauer spectroscopy^20^, extended X-ray absorption fine structure spectroscopy^21^,^22^ and neutron scattering^23^,^24^. An ideal annealing recipe would solely evacuate apical oxygen atoms while keeping regular oxygen sites at the O(1) and O(2) sites occupied.

## Results

In this study, we used thin film Pr_2_CuO_4_ samples (1000 Å thick) synthesized by state-of-the-art molecular beam epitaxy (MBE). In contrast to bulk samples, the large surface-to-volume ratio of thin films along with their tenuity itself is advantageous in achieving homogenous oxygen configurations. Thin films of Pr_2_CuO_4_ have been grown intentionally at temperatures lower than optimal as a reduced crystallite dimensions are advantageous for a homogeneous annealing experience.

In general the annealing process in oxide materials is a diffusion process. In particular, regular O(1) and O(2), as well as apical O(3) sites are occupied or evacuated in the T′-cuprates. Here, we use a high precision partial oxygen pressure monitoring and control system (POPMCS) combined with X-ray diffraction and transport data of Pr_2_CuO_4_ for the analysis of the annealing process. A commercial quartz tube furnace equipped with a turbo molecular pump and POPMCS was used. The Pr_2_CuO_4_ film was mounted on the tip of a SSA-S alumina tube placed at the center of the quartz tube.

**Figure 1:**
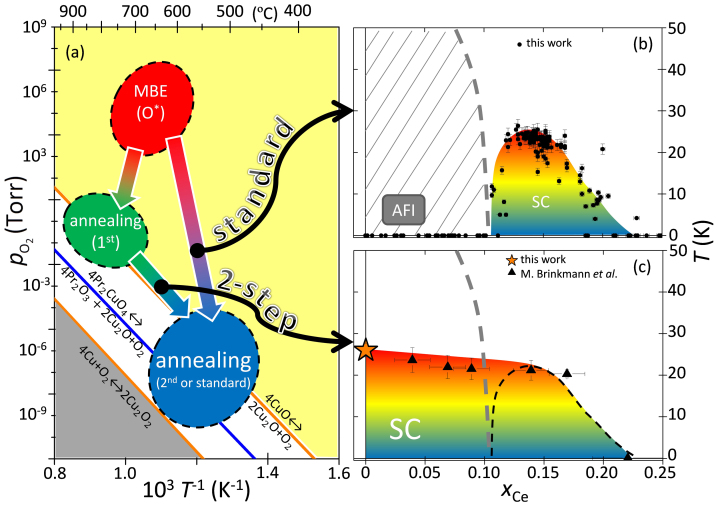
Annealing paths of Pr_2_CuO_4_ and the resulting electronic phase diagrams. In (a), the thermodynamic phase diagram is plotted where logarithmic and reciprocal scaling is used for the oxygen pressure and the absolute temperature, respectively. Thermodynamic stability lines for the copper-oxygen system and Pr_2_CuO_4_^52^ are shown. Pr_2_CuO_4_ films were grown using a radio-frequency activated oxygen plasma (O*) by molecular beam epitaxy. The oxygen pressure during the synthesis is 2 × 10^−6^ Torr, corresponding to an equilibrium molecular oxygen pressure of 10^6^ Torr. The synthesis temperature of Pr_2_CuO_4_ is 650–750°C. Standard annealing is carried out at temperatures between 550 and 650°C under 10^−9^ Torr. In the two-step annealing process^53^, Pr_2−x_Ce_x_CuO_4_ is annealed *ex situ* first at 750–850°C and 7.6 × 10^−2^ Torr O_2_ and subsequently annealed at temperatures between 450 and 700°C under high vacuum. In (b), the doping dependence of the superconducting phase diagram of Pr_2-x_Ce_x_CuO_4_ is shown for 273 different samples obtained by the standard annealing process. For 0.00 < *x* < 0.10, Pr_2-x_Ce_x_CuO_4_ is an antiferromagnetic insulator (AFI). For 0.11 < *x* < 0.23, superconductivity is induced by the standard annealing process with a maximum *T*_c_ of 25 K at *x* = 0.14. In (c), the doping dependence of the superconducting phase diagram of Pr_2-x_Ce_x_CuO_4_ is shown. Data points (black triangle) have been taken from^16^. At *x* = 0.00, results of 84 samples are summarized (star). Dashed lines represent the phase diagram as obtained in Fig. 1(b).

Starting from the standard annealing process (Fig. 1) typically applied to electron doped cuprates^25^,^26^, we split the annealing procedure, thus a two-step annealing process. Our systematic investigation on this new two-step annealing scheme reveals that only certain annealing conditions are suitable to preserve the T′-structure and induce superconductivity into Pr_2_CuO_4_. In Fig. 1b, we plot the electronic phase diagram of Pr_2-x_Ce_x_CuO_4_ (standard annealed), where the superconducting transition temperatures of 273 *c*-axis oriented, single phase thin films of Pr_2-x_Ce_x_CuO_4_ on (001) SrTiO_3_ (a = 3.905 Å) substrates are shown for 0.00 < *x* < 0.25. In contrast to the phase diagram for standard annealed Pr_2-x_Ce_x_CuO_4_, the *ex-situ* two-step annealing process allows superconductivity even without cerium. The phase diagram shown in Fig. 1c shows that superconductivity appears at all doping levels up to *x* ≈ 0.22 and the highest *T*_c_ is not at *x* = 0.15 but 0.00, in stark contrast to the commonly observed phase diagram (Fig. 1b). The newly obtained superconducting phase diagram indicates that the apparent symmetry of electronic phases for hole and electron doped cuprate superconductors with respect to the antiferromagnetic-insulating ground state might be an artifact of commonly used annealing treatments, thus, not representative. Instead, it appears that for zero doping, only the T-phase is an antiferromagnetic Mott insulator whereas the T′-phase is a superconductor, in agreement with the first principle methods' predictions^2^,^3^,^4^.

## Discussion

**Figure 2:**
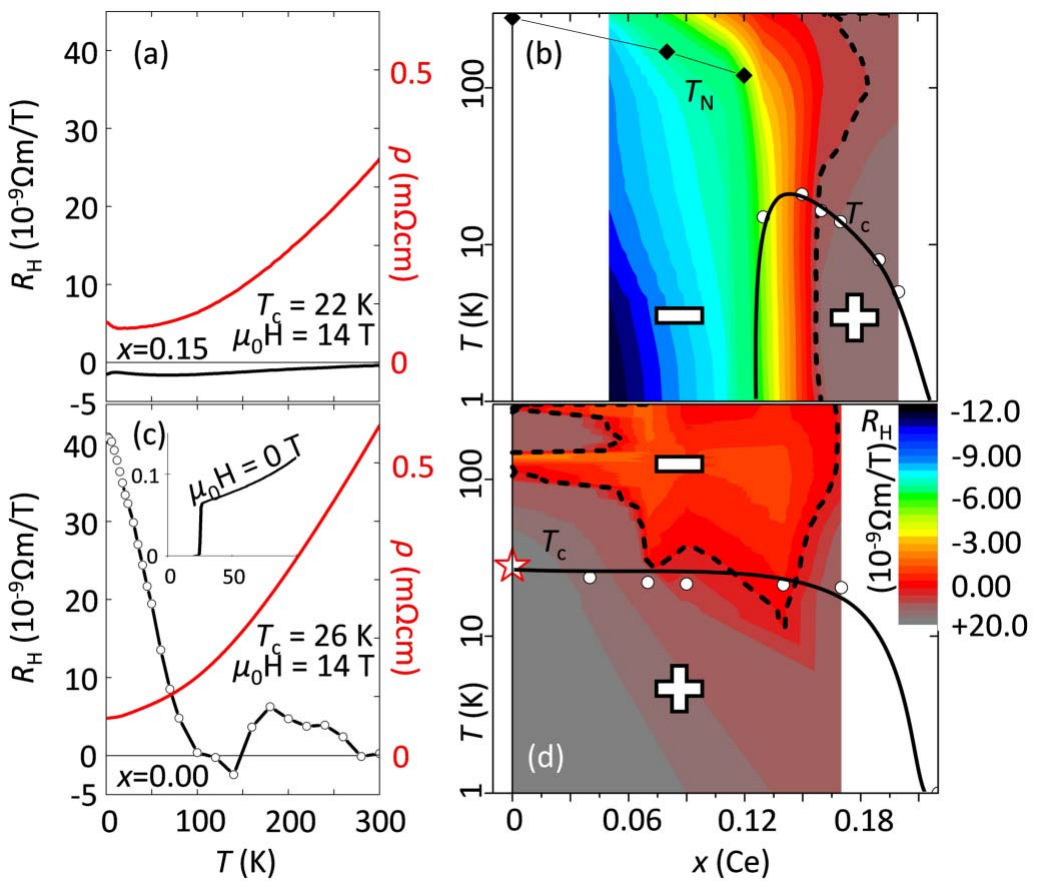
For the standard annealing process (a) and (b), the highest *T*_c_ is observed for *x* = 0.15. In (a) the Hall coefficient *R*_H_ (black line) and the resistivity at μ_0_*H* = 14 T are plotted as a function of *T* for standard annealed Pr_2-x_Ce_x_CuO_4_ with *x* = 0.15 at μ_0_*H* = 14 T and in (b) *R*_H_ is plotted as a function of *T* and *x* (data taken from^13^,^54^,^55^). Additionally, *T*_N_ and *T*_c_ are plotted as a function of *x* (*T*_N_ taken from^37^ and *T*_c_ from^13^). The “+” and “−” marks indicate the sign of the Hall coefficient *R*_H_ and are separated by the dashed line. The cross-over from “+” to “−” is at *x* ≈ 0.165 and coincides with the quantum critical point^56^. In case of conventionally annealed Pr_2-x_Ce_x_CuO_4_ the Hall coefficient develops monotonically upon electron doping (Ce doping) up to *x* ≈ 0.165. For the 2-step annealing process (c) and (d), the highest *T*_c_ is observed at *x* = 0.00 (star). In (c) *R*_H_ (black line) and the resistivity at μ_0_*H* = 14 T are plotted as a function of *T* for Pr_2_CuO_4_ treated by a 2-step annealing process at μ_0_*H* = 14 T and in (d) as a function of *T* and *x* (*T*_c_ data taken from^16^). The “+” and “−” marks indicate the sign of the Hall coefficient *R*_H_ and are separated (*R*_H_ = 0) by the dashed line. In case of 2-step annealed Pr_2_CuO_4_ the Hall coefficient is positive at 300 K, and at 150 K and 120 K, a sign change appears. Below 120 K, the Hall coefficient is positive down to 1.7 K. Upon electron doping (Ce doping) the low temperature Hall coefficient stays positive irrespective of the Ce concentration level. The contour-plots (b) and (d) were made from linear interpolation of *R*_H_(*T*) curves for *x* = 0.05, 0.075, 0.09, 0.10, 0.12, 0.14, 0.15, 0.17, 0.19 and *x* = 0.00, 0.06, 0.08, 0.10, 0.15, 0.17 in (b) and (d), respectively.

**Figure 3:**
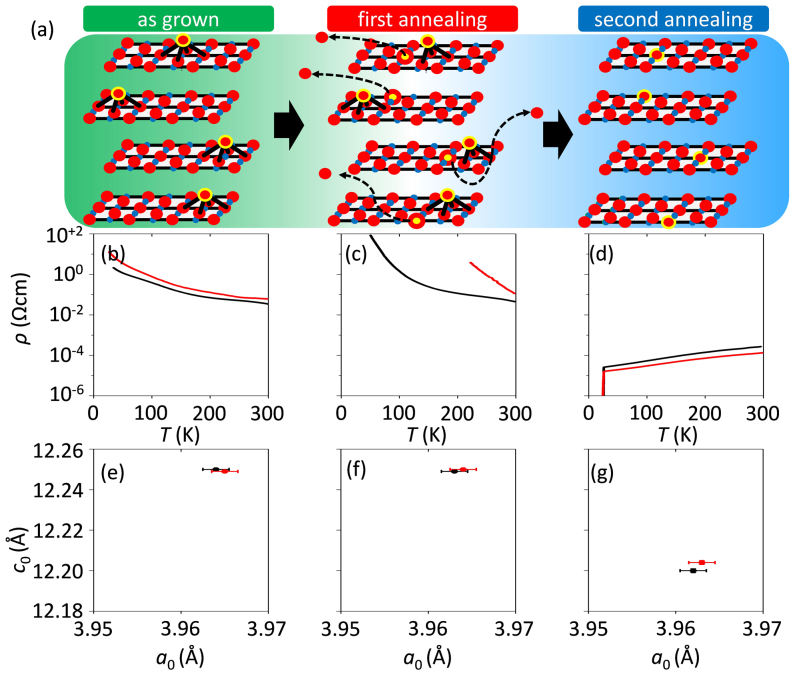
Schematic and simplified copper-oxygen configurations of the CuO_2_ planes in Pr_2_CuO_4_ (square planar coordinated cuprates) in accordance to the results deduced from neutron and X-ray scattering analysis and electronic transport data. (a) In the as-grown state, random apical sites of copper are occupied (apical oxygen). During the first annealing step of our two-step annealing procedure, not apical but regular oxygen sites of the CuO_2_ planes are being evacuated. During the second annealing step, the defective CuO_2_ plane is being “healed” by an oxygen rearrangement from the apical sites to regular in-plane sites (shrinkage of c-axis). (b)–(d) Evolution of *ρ*(*T*) characteristics and lattice constants after each synthesis step. The as-grown T′-Pr_2_CuO_4_ thin film is insulating and the optimally reduced films (after step II) are superconducting while *ρ*(300 K) is reduced by more than 2 orders of magnitude. The T′-Pr_2_CuO_4_ thin films just after step I are even less conductive than the as-grown ones. (e)–(g) The in-plane lattice constant (*a*_0_) remains constant throughout the annealing process while that of *c*-axis (*c*_0_) shows an abrupt drop after step II. The lattice parameters *a*_0_ and *c*_0_ have been estimated from a Nelson-Riley function of the (*h*03*h*) and (002*l*) reflections, respectively.

Comparing the influence of doping to the influence of annealing to Pr_2_CuO_4_ reveals that a hidden, hole-like Fermi surface may be present. The Ce doping dependence of the evolution of the Fermi surface of Nd_2-x_Ce_x_CuO_4_ has been reported by Armitage *et al.*^27^ for *x* = 0.04, *x* = 0.10 and *x* = 0.15. Traces (small but finite density of states) of a hole-like Fermi surface can be detected even for *x* = 0.04 27. However, such a sample is neither metallic nor superconducting owing to the annealing conditions applied. The hidden Fermi surface suggests that the applied annealing conditions were not optimal. Commonly, the observed Hall coefficient^14^ is negative for *x* = 0.04. The negative Hall coefficient can be attributed to “hot spots” located at (π, 0) and (0, π). The overall contribution to the Hall coefficient of those hot-spots is significant for Pr_2-x_Ce_x_CuO_4_ as the Hall coefficient is negative up to *x* ≈ 0.17 (Fig. 2b). The Hall coefficients *R*_H_ taken on superconducting Pr_2_CuO_4_ show unambiguously that the origin of metallic conduction and superconductivity itself is not electron doping but points towards a redistribution of spectral weight from those anti-ferromagnetic “hot-spots” into the hole-like Fermi-surface^25^. Figure 2(c) shows the temperature dependence of *R*_H_ for superconducting Pr_2_CuO_4_, and the Hall coefficient of standard annealed Pr_2-x_Ce_x_CuO_4_ is shown in Figs. 2a and b. For standard annealed Pr_2-x_Ce_x_CuO_4_, the highest *T*_c_ is at *x* = 0.15 and those samples have a negative *R*_H_, while *R*_H_ is positive in superconducting Pr_2_CuO_4_. Moreover, the Hall coefficient remains positive even after cerium doping (Fig. 2d) when an elaborate annealing process has been applied^16^. In general, the Hall conductivity of a metal is expressed^28^,^29^,^30^ as a function of the Fermi topology *dε*(***k***)/*d****k*** and the anisotropic relaxation time *τ*(***k***) at Fermi surface. The different signs of *R*_H_ observed for the superconducting Pr_2_CuO_4_ and the standard annealed superconducting Pr_2-*x*_Ce_x_CuO_4_ emphasize that superconductivity in Pr_2_CuO_4_ is induced via another route than doping. In other words, the role of the 2-step annealing is not that of doping electron carriers via possible formation of oxygen vacancies at the regular oxygen sites. Moreover, the positive Hall coefficient of superconducting Pr_2_CuO_4_ reflects the situation of a hole like Fermi surface which develops upon removal of apical oxygen, which is in contrast to the hole-doped analogues, where both, the oxygenated T-La_2_CuO_4 + δ_ and optimally Sr-doped La_1.85_Sr_0.15_CuO_4_ have positive *R*_H_^31^,^32^: the additional oxygen acts as a dopant for holes and in a similar way as Sr doping. We like to highlight the fact that the temperature dependence of the Hall coefficient is not that of a simple metal but rather demonstrates the competition between a hole-like metal and an antiferromagnetic insulator. This asymmetric scenario^1^,^33^,^34^,^35^ between square- and octahedral coordinated cuprates also shows that their electronic correlations are entirely different^3^. The absence of a doping mechanism in our elaborate annealing process is independently supported by the fact that the in-plane lattice constants of as-grown and annealed Pr_2_CuO_4_ films are constant upon annealing as it is well known that electron-doping stretches and hole-doping shrinks the Cu-O bonds in the CuO_2_ planes due to accumulation or depletion of electrons to/from the Cu-O 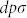 anti-bonding bands^36^. The presence of additional oxygen in as-grown Pr_2_CuO_4_ is well established^23^,^24^ as is its removal by annealing. We visualized our annealing scenario in Fig. 3a. The as-grown crystal contains more than the stoichiometric amount of oxygen which are randomly distributed at apical sites (Fig. 3a). After the first annealing step we find that the lattice parameters are nearly unchanged (Fig. 3f) when compared to the as-grown sample (Fig. 3e). However, its resistivity value is significantly higher (Fig. 3c). We explain such behavior by the introduction of oxygen vacancies in the CuO_2_ plane since such defects would disturb electronic conduction severely. The second annealing step does repair those in-plane defects by relocating apical oxygen atoms to the planes and consequently the resistivity is lowered significantly (Fig. 3d). This final step creates a situation similar to what has been observed after an annealing treatment^37^ for the cerium doped superconductors^38^. Overall we do observe that the *c*-axis lengths decreases upon annealing (Fig. 3 e–g) and that has been unambiguously proven to be associated to the removal of apical oxygen by neutron scattering^23^,^24^. A typical value of the oxygen off-stoichiometry estimated from neutron scattering experiments of as-grown Nd_2_CuO_4 + δ_ single crystals is δ ≈ 0.05, which indicates that one Cu ion out of ten unit cells is pyramidal coordinated. Experimentally, this is a sufficient condition to stabilize a long-range antiferromagnetic order even at Ce doping levels of *x* = 0.15^39^. In Ref. 39 it was shown that even for *x* = 0.15 the as-grown cuprate is an antiferromagnetic insulator with a *T*_N_ = 150 K. After annealing, however, the cuprate system goes into the superconducting state. The only chemical difference is that occupied apical oxygen sites have been evacuated during that annealing process. Those occupied apical oxygen sites break the symmetry for all nearest and next-nearest-neighbor Cu plaquettes. Such a locally broken symmetry localizes electrons primarily on one Cu site and induce a gap in the Fermi surface. Therefore, the doping process in electron doped cuprates might be considered as a band filling process, as its ground state is already a metal^40^.

**Figure 4:**
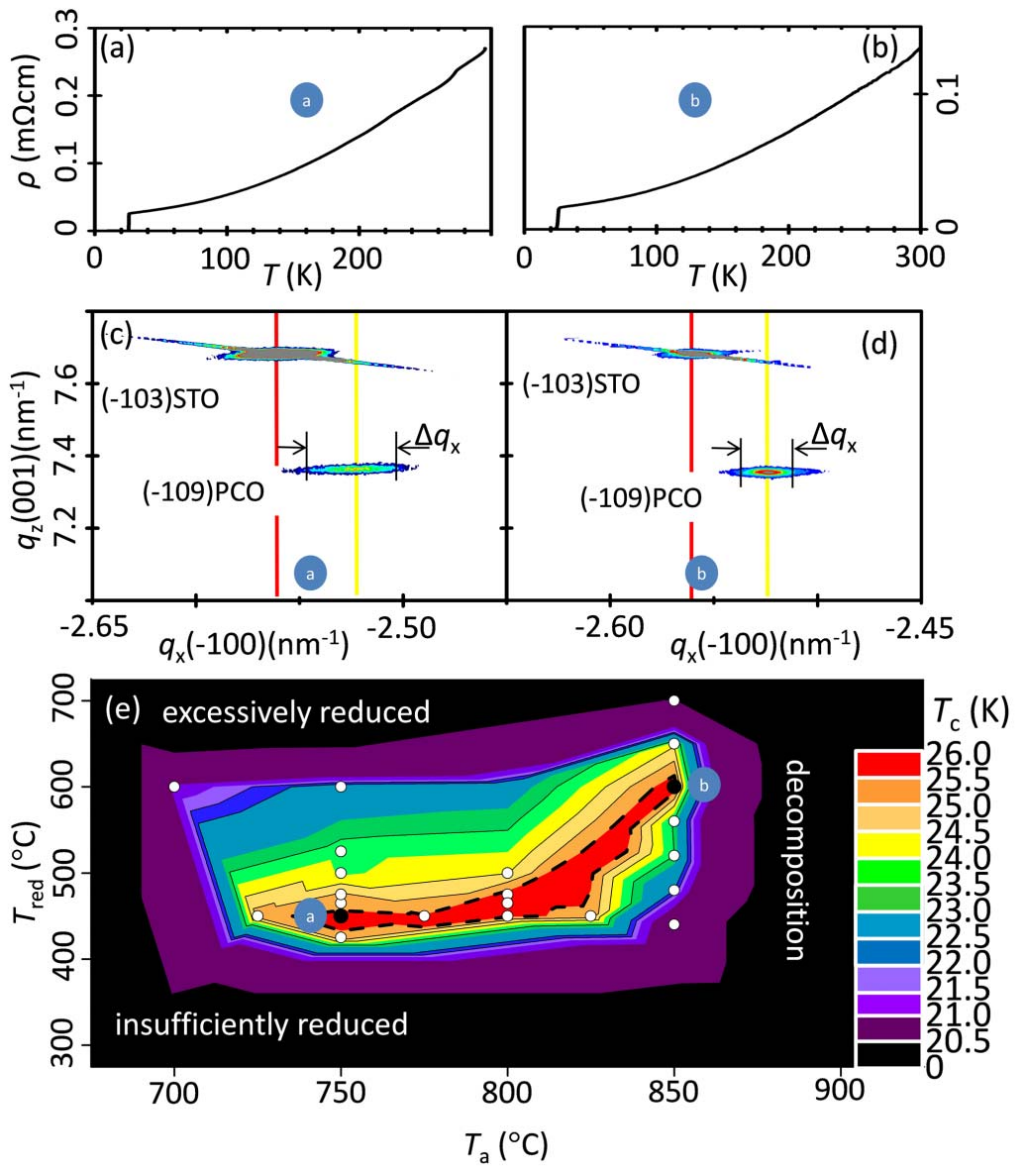
The temperature dependence of the resistivity (a,b), their associated high resolution reciprocal space maps (HRRSM) of fully relaxed Pr_2_CuO_4_ films grown on (001)SrTiO_3_ substrates (c,d), and the relationship between the first (*T*_a_) and second (*T*_red_) annealing temperature and their influence on the superconducting transition temperature *T*_c_ (e). In (a,c), a Pr_2_CuO_4_ film was annealed at *T*_a_ = 750°C and 7.6 × 10^−2^ Torr oxygen for 1 h (first annealing step), followed by a reduction process at *T*_red_ = 450°C under high vacuum for 10 min. The electronic transport shows metallic behavior with a superconducting transition at 26.0 K and a residual-resistivity-ratio (RRR) = 7. The relative position of the (−109) diffraction spot of Pr_2_CuO_4_ to the (−103) SrTiO_3_ diffraction spot shows that Pr_2_CuO_4_ films are epitaxial but relaxed grown on (001) SrTiO_3_. The in-plane lattice constant of the Pr_2_CuO_4_ films is 3.96 Å. (Δ*q*_x_)^−1^ ≈ 80 nm provides a rough estimation of the lateral crystallite dimensions. In (b, d), a Pr_2_CuO_4_ film was annealed at *T*_a_ = 850°C and 7.6 × 10^−2^ Torr oxygen for 1 h (first annealing step), followed by a reduction process at *T*_red_ = 650°C under high vacuum for 10 min. The electronic transport shows metallic behavior with a superconducting transition at 25.0 K and RRR > 5. The relative position of the (−109) diffraction spot of Pr_2_CuO_4_ to the (−103) SrTiO_3_ diffraction spot shows that Pr_2_CuO_4_ films are epitaxial but relaxed grown on (001) SrTiO_3_. The in-plane lattice constant of the Pr_2_CuO_4_ films is 3.96 Å. (Δ*q*_x_)^−1^ ≈ 250 nm provides a rough estimation of the lateral crystallite dimensions. The influence of the annealing history on the superconducting transition temperature *T*_c_ is given in (e). Here, the oxygen partial pressures during the first and second annealing steps were kept constant and are 7.6 × 10^−2^ Torr and high vacuum, respectively. *T*_c_ levels as high as 26.0 K can be reached for Pr_2_CuO_4_ films grown on (001) SrTiO_3_ substrates.

It is worth mentioning that the entire annealing process is a diffusion process as long as thermodynamic limits are not violated. Certainly, those limits have been violated considering earlier reports^41^. In contrast to the standard annealing process applied for bulk specimens, thermodynamic constraints, e.g., the Pr_2_CuO_4_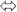 Pr_2_O_3_ + Cu_2_O stability line, may not be crossed in our 2-step annealing process. As for the standard annealing process, reduction conditions below the thermodynamic stability regions may harm the T′ phase, therefore RE_2_O_3_ oxides are often observed and consequently cause an increase of the absolute resistivity value. The annealing conditions applied in the first annealing step of our experiments are above the thermodynamic stability lines of Pr_2_CuO_4_ and CuO, thus, decomposition products, i.e., Pr_2_O_3_, can be ruled out in contrast to other experiments as we do not see indication of their presence either by transmission electron microscopy or X-ray diffraction. Besides the influence of the annealing conditions on the electronic transport properties (Fig. 4a,b), the crystallite dimensions of the thin film are also affected. Low annealing temperatures result in larger (Δ*q*_x_)^−1^ values (Fig. 4c and 4d), though the superconducting transition temperatures are constant (Fig. 4a, 4b). Both of the annealing steps of our two-step annealing process are not independent and their correlation to superconductivity is visualized in Fig. 4e where the superconducting transition temperature (*T*_c_) is plotted as a function of the first- (*T*_a_) and second- (*T*_red_) annealing temperatures. For optimal superconducting transition temperatures, a low *T*_a_ requires a low *T*_red_ and a high *T*_a_ requires a high *T*_red_. Consequently, when the annealing time and the oxygen partial pressures are kept constant, optimal superconducting transition temperatures are associated to *T*_a_ and *T*_red_ in an arc shaped relation.

Finally, we compare our data to results reported from first principle calculations mentioned earlier^2^,^3^,^4^. The contrasting ground states in square-planar and octahedral coordinated cuprates, i.e., T′ and T, are consequences of the difference in the charge-transfer gap Δ_0_, originating primarily from the different oxygen coordination. Vacant apical sites substantially reduce the electrostatic potential at the copper site, thus, the 3*d*^9^ Cu energy levels of the T′-phase are lower than in the T-phase, whereas the 2*p*^6^ O energy levels remain almost constant^42^,^43^. A simple evaluation of the unscreened Δ_0_ from Madelung potential calculations^44^,^45^ show that the difference in Δ_0_ between T′- and T-phases is in the range of several eV – therefore, the charge transfer gap might be very small or may even vanish in the T′-cuprates. Under such circumstances, the model of ionic binding, which is tacitly assumed in the discussion of the charge-transfer energy, loses its vindicability. Instead, hybridization effects between Cu 3*d*_x_^2^_-y_^2^ and O 2*p*_xy_ orbitals may dominate electronic correlations, though they are not taken into account in the commonly used *t*-J model^46^. A superconducting ground state in square planar coordinated cuprates, where doping is not a prerequisite but an option, may promote a deep understanding of the rich variety of electronic phases of cuprates as they depend on coordination, doping and diluted impurities^47^. Moreover, the new phase diagram of square-planar coordinated cuprates implies the following question: Does *T*_c_ further increase upon hole-doping? A recent article by Takamatsu *et al.*^48^ indeed observed superconductivity in hole doped square-planar coordinated cuprates. Answering may provide a fundamental understanding of the mechanism of high temperature superconductivity. Certainly, the induction of a long range commensurate 3D antiferromagnetic order by a tiny amount of apical oxygen in T′-cuprates demand for a thorough analysis outside of the commonly successful theoretical treatments. As the competition of antiferromagnetic and superconducting order in T′-cuprates ultimately tunes the electronic properties, e.g., *ρ*(*T*), *R*_H_(*T*), a microscopic understanding would be beneficial. The possible solution for a quantitative analysis of site specific occupancies of oxygen in T′-cuprates is either via neutron scattering experiments (bulk samples) or ^17^O nuclear magnetic resonance (NMR) spectroscopy^49^.

## Methods

Thin films of c-axis oriented, single phase Pr_2_CuO_4_ were epitaxially grown on (001) SrTiO_3_ (a = 3.905 Å) substrates by molecular beam epitaxy (MBE). The growth of the T′-Pr_2_CuO_4_ films was performed in a custom-designed MBE chamber^50^,^51^ (base pressure ~ 10^−9^ Torr) from metal sources by using multiple e-gun evaporators and an atomic oxygen source (0.5 sccm, radio-frequency (RF) power of 250 W) as an oxidizing agent. The cation stoichiometry was adjusted by controlling the evaporation beam flux of each constituent element by electron impact emission spectrometry (EIES) (Guardian IV, Inficon, USA) via feedback loops to the e-guns. Ultra-fine tuning of the evaporation beam fluxes (± 0.005 Å/s) was done by reflection high-energy electron diffraction (RHEED) monitoring^51^. Typically, the substrate temperature for the growth of T′-Pr_2_CuO_4_ thin films was *T*_s_ = 600–650°C. The film thickness is 1000 Å. For comparison purpose, some of the films were reduced *in-situ* after the growth under the ultra-high vacuum (UHV) environment.

Using the MBE-grown films, we investigated the reduction condition dependence of the properties of T′-Pr_2_CuO_4_. A commercial quartz tube furnace of 60 cm length and 30 mm diameter was used. The furnace is equipped with a turbo molecular pump (TMP) and a commercial (SiOC-200, STLAB, Japan) high precision partial oxygen pressure monitoring and control system (POPMCS). The POPMCS allows a precise control of the oxygen partial pressure between 10^−1^ to 10^−16^ atm by mixing an inert gas, e.g., N_2_, and oxygen at an electrochemically controlled oxygen diffusor (yttrium stabilized zirconium oxide). The Pr_2_CuO_4_ film was mounted on the tip of a SSA-S alumina tube placed at the center of the quartz tube in longitudinal direction. Prior to its first usage the quartz tube was cleaned in boiling piranha clean whereas the alumina tube was rinsed by deionized water. The cleaned quartz tube and SSA-S alumina tube were prebaked at 1000°C for 10 h under ultra-high vacuum. Prior to the first annealing step, the partial pressure of oxygen was adjusted to a defined value. The N_2_/O_2_ gas mixture was kept at a constant flow rate of 500 sccm throughout all experiments. The second annealing step is performed in the same tubular furnace evacuated in 10^−5^ Torr residual gas pressure.

## Author Contributions

All the MBE samples for *ex-situ* annealing experiments were prepared by H.Y. or Y.K. O.M., K.Y. and M.M. performed the *ex-situ* post-annealing experiments as well as most of the XRD, AFM, and *ρ*(*T*) measurements. *In-situ* annealing experiments were done by A.T. Y.K. carried out XRD experiments as well as characterization of magnetic properties by using a SQUID magnetometer. H.I. prepared the Hall bars and H.I. and Y.K. performed measurements of the Hall coefficient. M.N. along with other authors discussed the results and commented on the paper. All work was coordinated and overseen by H.Y. and M.N.

## Supplementary Material

Supplementary Information Supplementary information
