# Supplementary material for: Emerging superconductivity hidden beneath charge-transfer insulators
Source: Sci Rep. 2013 Jul 26;3:2235. doi: 10.1038/srep02235 (PMC3724181; doi:10.1038/srep02235)
Supplement: Supplementary Information [file srep02235-s1.doc]

Emerging superconductivity hidden beneath charge-transfer insulators

Yoshiharu Krockenberger^1^, Hiroshi Irie^1^, Osamu Matsumoto^2^, Keitaro Yamagami^1†^, Masaya Mitsuhashi^1†^, Akio Tsukada^1‡^, Michio Naito^2^, and Hideki Yamamoto^1★^

^1^NTT Basic Research Laboratories, NTT Corporation,

3-1 Morinosato-Wakamiya, Atsugi, Kanagawa 243-0198, Japan.

^2^ Department of Applied Physics, Tokyo University of Agriculture and Technology, 2-24-16 Naka-cho, Koganei, Tokyo 184-8588, Japan.

^†^ On leave from Nagaoka University of Technology.

^‡^ Present address: Department of Applied Physics, Tokyo University of Science, 1-3 Kagurazaka, Shinjuku, Tokyo 162-8601, Japan.

^★^ Correspondence and requests for materials should be addressed to Y. K. (e-mail: [yoshiharu.k@lab.ntt.co.jp](mailto:yoshiharu.k@lab.ntt.co.jp))

**Supplementary information**

Film growth methods.

Thin films of c-axis oriented, single phase Pr_2_CuO_4_ were epitaxially grown on (001) SrTiO_3_ (a = 3.905 Å) substrates by molecular beam epitaxy (MBE). The growth of the T’-Pr_2_CuO_4_ films was performed in a custom-designed MBE chamber (base pressure ~10^-9^ Torr) from metal sources by using multiple e-gun evaporators and an atomic oxygen source (0.5 sccm, radio-frequency (RF) power of 250W) as an oxidizing agent. The cation stoichiometry was adjusted by controlling the evaporation beam flux of each constituent element by electron impact emission spectrometry (EIES) (Guardian IV, Inficon, USA) via feedback loops to the e-guns. Ultra-fine tuning of the evaporation beam fluxes (± 0.005 Å/s) was done by reflection high-energy electron diffraction (RHEED) monitoring[^2^](#_ENREF_2). Typically, the substrate temperature for the growth of T’-Pr_2_CuO_4_ thin films was *T*_s_ = 600 - 650°C. The film thickness is 1000 Å. For comparison purpose, some of the films were reduced *in-situ* after the growth under the ultra-high vacuum (UHV) environment.

Characterization methods.

The reflection high-energy electron diffraction (RHEED) method was used as an *in situ* and real-time phase analysis technique. RHEED allows a thorough tuning of the stoichiometry and readily identifies impurity phases , thus, allows the synthesis of high quality Pr_2_CuO_4_ thin films. Here, we intentionally reduced the synthesis temperature of the Pr_2_CuO_4_ thin films in order to reduce the crystallite dimensions. This process is necessary in order to reduce the annealing times. Nonetheless, the Pr_2_CuO_4_ thin films were single phase and *c*-axis oriented. A powder diffractometer was used for the determination of the c-axis lattice parameter of Pr_2_CuO_4_ films and high-resolution reciprocal space maps (RSM) were taken by a Bruker AXS D8 Advanced four-circle diffractometer equipped with a two-bounce (220) Ge monochromator. The lattice parameters *a*_0_ and *c*_0_ of Pr_2_CuO_4_ films have been determined using a Nelson-Riley relation of the (*h*03*h*) and (002*l*) reflections, respectively. The error bars of the in-plane lattice and *c*-axis length estimated by a Nelson-Riley method are as small as 1×10^-2^ Å and 5×10^-3^ Å, respectively. Four silver electrodes were deposited on top of the Pr_2_CuO_4_ film for transport measurements. Resistivity measurements were carried out by using a standard four-probe method in a liquid Helium Dewar vessel.

*Ex-situ* annealing in vacuum tubular furnace.

Using the MBE-grown films, we investigated the reduction condition dependence of the properties of T’-Pr_2_CuO_4_. A commercial quartz tube furnace of 60 cm length and 30 mm diameter was used. The furnace is equipped with a turbo molecular pump (TMP) and a commercial (SiOC-200, STLAB, Japan) high precision partial oxygen pressure monitoring and control system (POPMCS). The POPMCS allows a precise control of the oxygen partial pressure between 10^-1^ to 10^-16^ atm by mixing an inert gas, e.g., N_2_, and oxygen at an electrochemically controlled oxygen diffusor (yttrium stabilized zirconium oxide). The Pr_2_CuO_4_ film was mounted on the tip of a SSA-S alumina tube placed at the center of the quartz tube in longitudinal direction. Prior to its first usage the quartz tube was cleaned in boiling piranha clean whereas the alumina tube was rinsed by deionized water. The cleaned quartz tube and SSA-S alumina tube were prebaked at 1000°C for 10 h under ultra-high vacuum. Prior to the first annealing step, the partial pressure of oxygen was adjusted to a defined value. The N_2_/O_2_ gas mixture was kept at a constant flow rate of 500 sccm throughout all experiments. The second annealing step is performed in the same tubular furnace evacuated in 10^-5^ Torr residual gas pressure.

Annealing procedure.

In the following, we describe the two-step annealing process. The first annealing step of Pr_2_CuO_4_ films was carried out at a constant annealing time *t*_a_ of 60 min. The annealing temperature *T*_a_ and the partial oxygen pressure *P*^a^_O2_ have been varied sample by sample. In the second annealing step, the tube furnace was evacuated (<10^-4^ Torr) and we systematically varied the reduction temperature *T*_red_ while keeping the annealing time *t*_red_ constant.

The thermodynamic stability boundary of Pr_2_CuO_4_ is a useful tool for tuning the annealing parameters in order to avoid decomposition products. Accordingly, the two-step annealing procedure is visualized within a thermodynamic phase diagram. In addition to the thermodynamic equilibrium lines for 4CuO ↔ 2Cu_2_O + O_2_, 2Cu_2_O ↔ 4Cu + O_2_, and Pr_2_CuO_4_ [^4^](#_ENREF_4), the equilibrium lines for atomic oxygen and molecular oxygen (O_2_ ↔ 2O*) are shown. The MBE growth conditions are located in the region of divalent copper though the decomposition line of Pr_2_CuO_4_ is in the stability region of monovalent copper. The large distance in the thermodynamic phase diagram between the synthesis and decomposition regions allows a careful tuning of the annealing parameters.

**Figure S1**. Synthesis route of the superconducting Pr_2_CuO_4_ films in the thermodynamic phase diagram. The stability lines of CuO and Cu_2_O, along with the equilibria oxidizing potential lines for atomic and molecular oxygen have been calculated using a commercial (MALT2, Kagaku Gijutsu-sha, Japan) thermodynamic database. The growth and annealing processes are typically carried out at *p*_O2_(*T*) conditions in the vicinity of divalent copper (hatched area in the log(*p*_O2_)(*T*^-1^) phase diagram). The stability line of Pr_2_CuO_4_ is also shown [^4^](#_ENREF_4), and lies in between the border lines of the two different copper valences (Cu^0^ and Cu^2+^). Phase formation of T'-Pr_2_CuO_4_ is achieved above the Cu^2+^ stability line, while the reduction process is performed below that line.

The influence of the annealing temperature *T*_red_ during the second annealing step on the resistivity *ρ*(*T*) behavior of Pr_2_CuO_4_ films grown and annealed (step I) under identical conditions, *T*_s_ = 600°C, *T*_a_ = 850°C and *P*^a^_O2_ = 2 × 10^-3^ atm are shown in Figure S2(a,b). An increase of *T*_red_ from 400°C to 650°C results in an increase of *T*_c_ and a decrease of the resistivity value. Any further increase of *T*_red_ up to 700°C causes an abrupt increase of resistivity value and superconductivity vanishes. Similar experiments have been carried out for films grown on (110) DyScO_3_ substrates [^5^](#_ENREF_5), where specimens were prepared mainly by solid state epitaxy. Figure S2(e) shows a clear influence of *T*_red_ on the *c*-axis lattice constant. When *T*_red_ is between 440 and 600°C, the *c*-axis length is *c*_0_ = 12.24 Å. For *T*_red_ higher than 600°C, the c-axis length shrinks to 12.22 Å and 12.20 Å for *T*_red_ = 650°C and 700°C, respectively. Next, we examined the influence of *T*_a_ in step I on the electronic transport properties (Fig. S2c). The transport properties of the T’- Pr_2_CuO_4_ films annealed at *T*_a_ = 850°C are optimal when *T*_red_ ~ 650°C [^5^](#_ENREF_5), whereas *T*_red_ ~ 475°C is optimal when *T*_a_ = 750°C. In particular, *T*_c_ becomes highest for *T*_red_ = 450°C ~ 475°C and *ρ*(300 K) lowest for *T*_red_ ~ 525°C when *T*_a_ = 750°C (Fig. 4). Note that the scattering of *T*_c_ and *ρ*(300 K) at a given *T*_a_ and *T*_red_ are consequences of thitherto unconsidered sample synthesis conditions. We may conclude that the optimal *T*_red_ in step II is predominantly governed by the annealing temperature *T*_a_ in step I. The difference in the optimal *T*_red_ for samples treated at *T*_a_ = 750°C and *T*_a_ = 850°C during step I, is as large as 200°C whereas the resulting *T*_c_ values are constant irrespective of *T*_a_. The Pr_2_CuO_4_ films now show a low resistivity value *ρ*(300 K) ≤ 300 μΩcm and high transition temperature of *T*_c_ ~ 25 - 26 K. A strong diamagnetic signal observed for the films annealed at *T*_a_ = 750°C in in-plane magnetic field configuration (Fig. S2(d)) reveals a bulk-like superconducting response. Therefore, scenarios like interface or filamentary superconductivity can be excluded. For comparison, the results of in situ UHV annealed Pr_2_CuO_4_ films are shown in Fig. S2(f).

Thermodynamic constraints of the two-step annealing procedure

**Figure S2.** Temperature dependence of resistivity (a-c), and magnetization (d) in Pr_2_CuO_4_ thin films after two-step annealing, as well as the associate c-axis length as a function of the annealing temperature *T*_red_ in the second annealing step. In (f), the temperature dependence of Pr_2_CuO_4_ films after *in-situ* annealing treatment are shown. Samples shown in (a-e) were grown at *T*_s_ = 650°C and annealed *ex situ* at *T*_a_ = 850°C for *t*_a_ = 60 min and *P*^a^_O2_ = 2 × 10^-3^ atm. Zero-field cooled and field-cooled magnetization data are shown for a Pr_2_CuO_4_ film with *T*_red_ = 630°C (d). Upon increasing *T*_red_, the c-axis length monotonically decreases and superconductivity is only observed in the limited area marked by “SC” (e). When *T*_red_ is between 440°C and 600°C, the c-axis length is between *c*_0_ = 12.24 and 12.23 Å. For *T*_red_ higher than 600°C, the c-axis length shrinks to 12.22 Å and 12.189 Å for *T*_red_ = 650°C and 700°C, respectively. For *c*-axis values of 12.22 Å, the superconducting (“SC”) transition temperature is highest. Mind, that the error bar of the *c*-axis length at *T*_red_ = 700 °C is larger compared to other temperatures as such annealing conditions are excessive and the X-ray diffraction peaks become rather broad. Films grown at *T*_s_ = 690°C and reduced in the UHV chamber (*P*_O2_ << 1 × 10^-8^ Torr) (f). The reduction temperature (*T*_red_) and duration (*t*_red_) are as follows: (1) *T*_red_ = 630°C, *t*_red_ = 10 min, (2) *T*_red_ = 625°C, *t*_red_ = 1h, (3) 1^st^: *T*_red_ = 625°C, *t*_red_ = 1h; 2^nd^: *T*_red_ = 600°C, *t*_red_ = 13h, and (4) *T*_red_ = 630°C, *t*_red_ = 9h. Unlike our electron-doped Pr_1.86_Ce_0.14_CuO_4_ films, prepared by an identical procedure [^6^](#_ENREF_6), the Pr_2_CuO_4_ films do not show any trace of superconductivity by *in situ* UHV annealing.

Optimal reduction conditions

So far we presented methods and procedures which allow an induction of superconductivity to Pr_2_CuO_4_ thin films. The annealing procedure by itself is a diffusion process where oxygen atoms move in the crystal. It is well known that the oxygen diffusion in anisotropic materials, i.e. Pr_2_CuO_4_, is also anisotropic[^7^](#_ENREF_7). In addition one has to not only consider the various environments of the different oxygen sites but also that certain oxygen atoms change sites whereas others simply diffuse. Utilizing the competition of those diffusion processes eventually allow the stabilization of a nearly defect-free oxygen configuration where oxygen atoms are located exclusively at O1 sites (the CuO_2_ planes) and the O2 sites (not directly above the Cu). While for bulk materials such a process can be traced by neutron scattering experiments, the small mass of thin films excludes this possibility. However, the existence of defects, i.e., occupied O3 sites, trigger a modification of the crystallographic unit cell dimensions. While a direct determination of the oxygen content of thin films can be ruled out, it is rather straightforward to determine the unit cell dimensions. Moreover, the X-ray diffraction peak width simultaneously provides information of the crystalline quality, i.e., cation disorder. We have used such measures in order to determine thermodynamic constraints of Pr_2_CuO_4_. While one limit is clearly defined by the decomposition of Pr_2_CuO_4_ into its simple oxides the other limit is given by the absence of superconductivity. From Fig. S3

**Figure S3**. The c-axis dependence of the superconducting transition temperature *T*_c_ of Pr_2_CuO_4_. Superconductivity (colored area) appears for 12.195 < *c* < 12.225 Å.

one can identify two distinguished areas where superconductivity does not appear. We would like to emphasize that the superconducting area in Fig. S3 does not represent a phase diagram but rather a distinct point of the electronic phase diagram of cuprates. In short, if Pr_2_CuO_4_ is to be annealed that the resulting c-axis lengths are between 12.195 and 12.225 Å, the sample will be superconducting.

Competing ground states in T’-Pr_2_CuO_4_

A situation, where the insulating as well as the superconducting Pr_2_CuO_4_ can be *selectively* prepared by any synthesis method, might be advantageous. From our present study, we may provide a general route to prepare superconducting Pr_2_CuO_4_ thin films as follows. (1) Growth of epitaxial Pr_2_CuO_4_ films with a thickness of < 100 nm. As we mentioned earlier, the crystalline quality of the as-grown Pr_2_CuO_4_ films was intentionally reduced in order to keep the annealing times in an accessible range. Any growth method, such as MBE, pulsed laser deposition (PLD), sputtering, and chemical vapor deposition (CVD) are promising methods since specific restrictions on the crystallinity of the as-grown Pr_2_CuO_4_ films are not required. (2) Annealing of the epitaxial films at *T*_a_ = 750°C ~ 850°C for 60 min under a mixture of flowing inert gas and O_2_ with *P*^a^_O2_ = 9 × 10^-5^ ~ 2 × 10^-3^ atm. The partial pressure of oxygen *P*^a^_O2_ should be lower (higher) for lower (higher) *T*_a_. (3) Successive annealing of the samples at lower *T*_red_ for 10 min under vacuum (*P*^red^ ≤ 1 × 10^-4^ Torr). The optimal *T*_red_ may vary over a temperature range between 400°C ~ 650°C, depending on the microstructure (grain size) of the films. After the second annealing step, samples should be quenched to room temperature under vacuum. This procedure allows to selectively synthesize either superconducting or insulating T’-Pr_2_CuO_4_.

Figure S4. The phase diagram of Pr_2-x_Ce_x_CuO_4+δ_ where the superconducting and the antiferromagnetic-insulating phases are shown as a function of doping, i.e., Ce doping, and as a function of the excess oxygen δ. The red-dashed line represents the excess oxygen content obtained by the standard annealing process. The blue-dashed line indicates the excess oxygen content obtained by a two-step annealing process. The grey shaded area represents the antiferromagnetic-insulating phase whereas the colored area the superconducting phase.

Since both phases, superconducting and antiferromagnetic insulating, can be induced in the same crystal by oxygen engineering, the phase diagram of electron doped cuprates has been modified into a 3 dimensional phase diagram, where doping *x* and the oxygen off-stoichiometry parameter δ are the variables. Typical synthesis and annealing recipes are along the red-dashed line. The relation between δ and *x* is triggered by an increasing amount of crystalline defects due to the Cerium incorporation. At low Cerium concentrations, the standard annealing process is insufficient to effectively reduce δ to zero. The two-step annealing process however, promotes an effective evacuation of O(3) sites. Since superconductivity is observed irrespective of the doping level, the commonly observed phase diagram of electron doped cuprates may be an artifact of the oxygen engineering process, itself.

Comparison with angle resolved photo emission spectroscopy (ARPES)

The Ce doping dependence of the evolution of the Fermi surface of Nd_2-x_Ce_x_CuO_4_ has been reported by Armitage *et al.* [^8^](#_ENREF_8) for *x* = 0.04, *x* = 0.10 and *x* = 0.15. For Pr_2-x_Ce_x_CuO_4_ with *x* = 0.04, Brinkmann *et al.* [^9^](#_ENREF_9) reported superconductivity after the sample has been treated by an improved annealing process. Traces (finite density of states) of a hole-like Fermi surface can be detected for *x* = 0.04 [^8^](#_ENREF_8). The apparent absence of a Fermi surface suggests that the applied annealing condition were not optimal. Commonly, the observed Hall coefficient [^10^](#_ENREF_10) is negative for *x* = 0.04. The negative Hall coefficient can be attributed to those “hot spots” located at (π, 0) and (0, π). However, such samples are neither metallic nor superconducting owing to the annealing conditions applied.

References
